# Seed germination ecology of endangered plant *Horsfieldia hainanensis* Merr. In China

**DOI:** 10.1186/s12870-024-05208-z

**Published:** 2024-06-01

**Authors:** Xiongsheng Liu, Yufei Xiao, Yong Wang, Renjie Wang, Ronglin Huang, Huizi Liang, Yi Jiang, Ying Jiang

**Affiliations:** Guangxi Key Laboratory of Superior Trees Resource Cultivation, Guangxi Forestry Research Institute, Nanning, China

**Keywords:** Seed biological characteristics, Environmental factors, Germination traits, Seedling growth, Endangered causes, Breeding techniques

## Abstract

**Background:**

*Horsfieldia hainanensis* Merr., an indicator species of China’s humid tropical rainforests, is endangered due to difficulties with population regeneration. In this study, the biological characteristics and germination adaptability of the seeds were studied for the first time, in order to provide a basis for analyzing the causes of endangerment and strategies for the artificial cultivation of *H. hainanensis*. The effects of biological characteristics (population, arils, seed coat, seed weight, seed moisture content) and environmental factors (temperature, light, drought, substrate, burial depth) on seed germination and seedling growth of *H. hainanensis* were studied.

**Results and discussion:**

The fruits were found to be capsules containing seeds wrapped in a pericarp and fleshy aril, which provide protection and assist in seed dispersal, but also pose risks to the seeds, as the peel and fleshy aril can become moldy under high temperature and humidity conditions. There were significant differences in fruit morphology and germination characteristics among different populations, and the seed quality of populations in Niandian village, Daxin County, Chongzuo City, Guangxi Zhuang Autonomous Region was better. The arils significantly inhibited seed germination, the germination of large seeds was better, and seedling growth from medium seeds was superior. *H. hainanensis* seeds were sensitive to dehydration, and intolerant to drought and low temperature, which is typical of recalcitrant seeds. The seeds are suitable for germination on a moist substrate surface with good water retention and breathability at 30–35℃.

## Introduction

Seeds are the bridge between the parent and offspring in plants [1]. Seed germination is the basis for natural regeneration of plant populations, and the characteristics of seed germination affect seedling survival, plant fitness, and the expression of the life cycle, which are directly related to the ability of plant populations to regenerate and survive [2]. Seed germination is susceptible to a combination of biotic (such as animals, plants and microorganisms) and abiotic (such as temperature, light, and moisture) factors [3, 4], as well as seed quality, seed phenotype, seed appendages, and seed surface structure [5], any of which may affect seed germination strategies, and these strategies determine plant survival and reproduction [6]. Barriers to natural regeneration caused by difficulties with seed germination or seedling survival are an important cause of endangerment in plants [7, 8]. For example, the endangerment of *Widdringtonia whytei* [9], *Astragalus nitidiflorus* [10], *Elaeagnus mollis* [11], and *Camptotheca acuminata* [12] can be attributed to problems with seed germination and seedling growth because of habitat loss and climate change. Therefore, understanding the characteristics of seeds and germination in endangered plant species and exploring the factors that limit seed germination can help to explain the causes of endangerment and provide theoretical support for the reintroduction and domestication of endangered plants to reconstruct populations and retore the ecological balance in the area [13].

*Horsfieldia hainanensis* Merr., a member of the genus *Horsfieldia* Willd., is a tall tree with a narrow range of distribution, which is endemic to China, dioecious, and a marker species of humid tropical rainforests. The presence of this marker species is of great value when investigating the floristic composition of tropical rainforest, the geographical distribution of the species, and its ecological habitat [14]. The leaves and bark of *H. hainanensis* can be used in medicines, and with their high oil content, the seeds can be used as high-quality raw materials for industrial products such as anti-condensates and in health-care and cosmetics goods. Moreover, the wood can be used to produce high-end furniture and decorative merchandise due to its bright red color [14]. Recently, due to its own biological characteristics, human destruction, and unlawful tree felling, the *H. hainanensis* habitat has been severely fragmented, and some populations are in decline. Its populations are now intermittently distributed in Guangxi, Yunnan, and Hainan provinces in an “island” or “dotted” pattern, mostly in shaded and wet valleys at an altitude of 400–450 m. The species has been listed as a Class II protected plant in China and, with its extremely small populations, has become endangered [15]. It is therefore imperative to study the conservation biology of *H. hainanensis*.

As previously reported, the low quantities of seed in storage, low viability of seeds in soil seed banks, and seeds were high aggregated distribution around the mother tree, along with their susceptibility to high temperatures and humidity, susceptibility to insect and ant damage, and low seed germination percentage (GP) under natural conditions, has resulted in a low seed to seedling conversion rate and high early seedling mortality [15]. Therefore, the population regenerates at a slow rate, which is an important reason behind the endangerment of the species [15]. The biological and germination characteristics of *H. hainanensis* seeds have not been widely reported. Moreover, the biological characteristics and ecological adaptability of its fruits and the main factors that affect seed germination are unknown. In the field observation, we found that the *H. hainanensis* population was highly exposed to rock, and many fruits were lost water easily and were air dried when they fell on the rocks; moreover, the fleshy peel and arils grew mold easily in the high temperature and high humidity environment, which affected seed germination. Therefore, we speculated that the special fruit structure and rocky mountain environment of the population may be the main factors limiting its seed germination. In order to verify our hypothesis, we first investigated the biological characteristics of different populations by measuring their fruit morphological characteristics, then carried out experiments on the effects of seed biological characteristics and environmental factors on seed germination, and finally analyzed the main factors affecting seed germination. The study aimed to explore (i) the biological characteristics and ecological adaptability of *H. hainanensis* fruits; (ii) the limiting factors for seed germination and the relationship between these factors and the endangerment of this species; and (iii) the technology used to sow and breed *H. hainanensis*, so as to provide a theoretical basis for the protection, restoration, and artificial propagation of this species.

## Materials and methods

### Experimental materials

The fruits of *H*. *hainanensis* generally mature from April to May. In March 2021, a survey was conducted on the fruiting status of wild populations of this species distributed in Guangxi Zhuang Autonomous Region and Yunnan Province, China. Only three wild *H. hainanensis* populations distributed in Niandian Village, Daxin County, Chongzuo City, Guangxi Zhuang Autonomous Region (ND); Nonggang National Nature Reserve, Guangxi Zhuang Autonomous Region (NG); and Xishuangbanna Botanical Garden in Yunnan Province (XSBN) were normally fruitful (Table 1). Therefore, in May 2021, mature fruits from the ND, NG, and XSBN populations were collected. The fruits of each population were mixed evenly after collection and taken to the laboratory for immediate measurement of fruit morphology and weight. Subsequently, the fruits were spread in a cool and ventilated place and then collected for subsequent experiments until the fruits naturally cracked open. All collection activities were approved by local authorities and competent authorities. The mature seeds of *H*. *hainanensis* were identifed by Professor Yi Jiang from Guangxi Forestry Research Institute as belonging to the genus *Horsfieldia* in the family Myristicacea. A voucher specimen was deposited in the Herbarium of Guangxi Forestry Research Institute, with voucher number GXFI0004820.

**Table 1:** *H. hainanensis* fruit collecting locations

Population	Location	GPS coordinates	Altitude (m)	Average annual temperature (℃) ^a^	Annual rainfall (mm) ^a^	Soil type
ND	Niandian Village, Daxin County, Chongzuo City, Guangxi Zhuang Autonomous Region	22.751° N, 106.798° E	375	21.7	1332	Calcareous soil
NG	Nonggang National Nature Reserve, Guangxi Zhuang Autonomous Region	22.484° N, 106.942° E	163	22.2	1311	Calcareous soil
XSBN	Xishuangbannan Botanical Garden in Yunnan province	21.927° N, 101.252° E	566	22.1	1486	Lateritic soil

*Note*^a^ Data from the National Meteorological Information Center (http://www.nmic.cn/)

### Determination of the morphological characteristics of fruits and seeds

Fifty intact and healthy fruits and seeds were randomly selected from each population, and this was repeated five times. First, the mass of each fruit and seed was measured using an electronic balance with an accuracy of ± 0.001 g, and then the horizontal and vertical diameters of each fruit and seed were measured using Vernier calipers with an accuracy of ± 0.01 mm (the fruits and seeds of *H. hainanensis* are oval in shape).

To determine seed moisture content, 50 healthy seeds were randomly selected from each population, and this was repeated three times. The weight of each fresh seed was measured using an electronic balance with an accuracy of ± 0.001 g, and then the seeds were dried in an oven at 103 °C. The dry weight of each seed was then determined to calculate the seed moisture content (SMC) [16].

### Experimental design for seed germination

#### General seed germination test

Unless otherwise stated, all germination experiments were carried out according to the following procedure. Before sowing, the seeds were disinfected with 0.2% potassium permanganate solution for 5 min, rinsed with clean water, and soaked in distilled water for 48 h. The seeds were sown in a plastic box (length × width × height = 28 cm × 17.5 cm × 8 cm) containing a 5-cm thick layer of autoclaved sand. Each seed was sown with the hilum downward and gently pressed until the fine sand covered the seed to a depth of 1/3 of the vertical diameter of the seed. The spacing between seeds was about 3 cm, and a total of 30 seeds were sown in each plastic box. The boxes were placed into an artificial climate incubator (Ningbo Yanghui RDN-1000, Ningbo Yanghui Instrument Co., Ltd., Ningbo, China) with a daytime temperature of 30 °C (12 h), nighttime temperature of 25 °C (12 h), periodic light (3000 lx 12 h/day), and 80% relative humidity. Germination was considered to have occurred when the radicle broke through the seed coat and grew to 1/2 of the seed horizontal diameter. Seed germination was observed and recorded daily, and appropriate water supplementation was provided. The experiment ended after 40 days. Seeds from the ND populations were used in all experiments, although the seeds of all three populations (ND, NG, and XSBN) were used in the experiment on seed germination among different populations.

Four parameters, germination percentage (GP), germination energy (GE), mean germination time (MGT), and vitality index (VI), were selected to evaluate seed vitality [17, 18] and calculated as follows:

$${\rm{GP = }}{{{\rm{total}}\,{\rm{number}}\,{\rm{of}}\,{\rm{germinated}}\,{\rm{seeds}}} \over {{\rm{number}}\,{\rm{of}}\,{\rm{test}}\,{\rm{seeds}}}}{\rm{ \times 100\% }}$$

$${\rm{GE = }}{{{\rm{number}}\,{\rm{of}}\,{\rm{seeds}}\,{\rm{germinated}}\,{\rm{normally}}\,{\rm{within}}\,{\rm{25}}\,{\rm{days}}} \over {{\rm{number}}\,{\rm{of}}\,{\rm{test}}\,{\rm{seeds}}}}{\rm{ \times 100\% }}$$

$${\rm{MGT = }}\sum {\left( {{{\rm{n}}_{\rm{i}}} \times {{\rm{t}}_{\rm{i}}}} \right)} /\sum {{{\rm{n}}_{\rm{i}}}}$$

Where *n*_*i*_ is the number of seeds that germinated on Day *i* after sowing and *t*_*i*_ is the number of days after sowing.

$${\rm{VI = }}\sum {\left( {{{\rm{n}}_{\rm{i}}}{{\rm{t}}_{\rm{i}}}} \right)} \times \left( {{\rm{LR + SH}}} \right)$$

Where LR and SH refer to length of the root (cm) and seedling height (cm) at the end of the germination test, respectively (in this study, the LR is the total length of radicle and hypocotyl) [19].

At the end of the seed germination period, 10 seedlings were randomly selected from each plastic box for the measurement of LR and SH (all seedlings in boxes with less than 10 seedlings were measured). The roots, stems, and leaves of the seedlings were separated and placed in an oven for 0.5 h at 105 °C, followed by heating at 65 °C to a constant weight. The underground biomass (UB) and aboveground biomass (AB) of the seedlings were weighed using an electronic balance with an accuracy of ± 0.001 g.

#### The effect of seed biological characteristics on seed germination

**Fig. 1:**
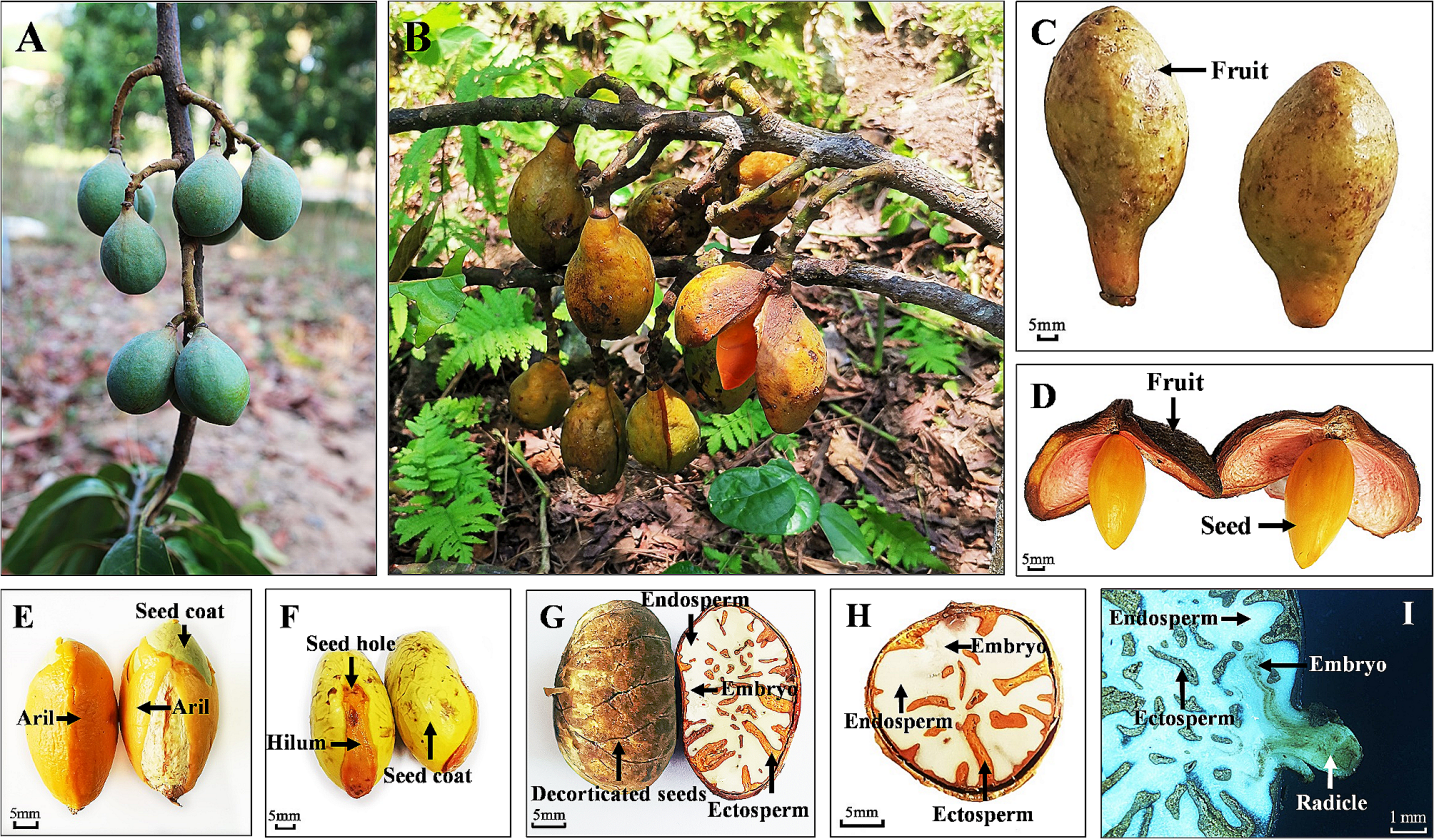
Morphological characteristics of the fruits and seeds of *H. hainanensis.***A**: Immature fruit. **B**: Mature fruit. **C**: Morphological characteristics of mature fruit. **D**: Seeds exposed after the fruit cracks open. **E**: Aril and seed coat. **F**: Seed morphological characteristics. **G**: Decorticated seed and longitudinal view of the seed interior. **H**: Transverse view of the seed interior. **I**: Longitudinal view of the seeds after germination

To compare differences in seed germination among the populations being investigated, 30 seeds were selected at random from each of the ND, NG, and XSBN populations, and this was repeated three times. To measure the effects of the aril and seed coat on seed germination, three seed treatments were established using the ND population: (i) seeds with aril and seed coat (WAS, Fig. 1E), (ii) decorticated seeds (DS, Fig. 1G), and (iii) seeds with the aril removed and seed coat retained (control, CK, Fig. 1F). Thirty seeds were randomly selected for each treatment, and this was repeated three times. Furthermore, 200 ND seeds were randomly selected and their arils removed. This was repeated three times, and the weight of each seed was measured using an electronic balance with an accuracy of ± 0.01 g (except for the seeds used to test the effects of the aril and seed coat on seed germination). The seeds were divided into five weight grades according to seed weight: ≤4.0 g, 4.0–5.0 g (including 5.0 g), 5.0–6.0 g (including 6.0 g), 6.0–7.0 g (including 7.0 g), and > 7.0 g. The number of seeds in each weight grade was recorded and the proportion of seeds in each weight grade was calculated. To investigate the effect of seed weight on seed germination, 30 seeds were randomly selected from each weight grade, and this was repeated three times. All experimental operations and conditions were the same as those described for the general seed germination test.

To investigate the desiccation tolerance of *H. hainanensis* seeds, fresh seeds from the ND population were selected and the initial weight (W1) measured. Next, the seeds were spread out and placed in a cool place indoors for natural dehydration to take place (during which the average daily temperature was 24.0–27.0 °C and the relative humidity was 70–85%). The weights of the seeds during this period (W2) were measured every 8 h (50 seeds were randomly selected each time, with three repeats, and weighed using an electronic balance with an accuracy of ± 0.001 g). The dehydration rate was calculated based on W1: Dehydration rate = (W1 − W2)/W1 × 100% [20]. The dehydration rates of the seeds were controlled at 5%, 10%, 15%, 20%, 25%, and 30%, to produce a total of six dehydration rates (an error of ≤ 0.5% was allowed). To test germination, 30 seeds were randomly selected from dehydration rate gradient and this was repeated three times. Fresh seeds (dehydration rate 0%) were used as controls. All experimental operations and conditions were the same as those described in the general seed germination test.

#### The effect of environmental factors on seed germination

The germination of *H. hainanensis* seeds in response to temperature was evaluated by incubating seeds at constant (15, 20, 25, 30, 35, and 40 °C) and fluctuating (30/20, 35/25°C) (12-h high/ 12-h low) temperatures (designed according to the temperature at the time of seed dispersal at the source of the *H. hainanensis* seeds). Thirty seeds were used per treatment, and each treatment was repeated three times. Except for the temperature conditions, all other experimental operations and conditions were the same as those described for the general seed germination test.

To determine the effect of light on seed germination, three light patterns, continuous light (3000 lx, 24/0 h light/dark), continuous darkness (0/24 h light/dark), and periodic light (3000 lx, 12/12 h light/dark), were set up. Thirty seeds were used per treatment, and each treatment was repeated three times. To ensure constant darkness, the seed germination rate under the continuous darkness treatment was recorded at the end of the germination period. Except for the light conditions, all other experimental operations and conditions were the same as those described for the general seed germination test.

To explore the effects of drought on seed germination, Polyethylene glycol-6000 (PEG-6000, Sigma-Aldrich, USA) solutions at concentrations of 0% (distilled water), 10%, 20%, and 30% were prepared. Thirty seeds were used in each of the four treatments, and each treatment was repeated three times. Two layers of filter paper were laid on the bottom of a plastic box and moistened with 15 mL of treatment solution. The seeds were then sown on the filter paper and placed in an incubator. The filter paper and PEG-6000 solution were changed every 2 days. All other experimental operations and conditions were the same as those described for the general seed germination test.

To investigate the effect of substrate on seed germination, fine sand (particle size 0.125–0.250 mm), clay loam (30, 40, and 30% sand, clay, and silt, respectively), peat soil (particle size 0–10 mm), and limestone soil (42, 25, and 33% sand, clay, and silt, respectively) were used. Thirty seeds were used in each of the four treatments, and each treatment was repeated three times. Fine sand, clay loam, and peat soil were purchased from Xuzhou Flame Commercial & Trading Co., China in April 2021. The limestone soil was obtained from the 0–20 cm soil layer at the collection location for the ND population and transported back to the laboratory. After air-drying for 7 days, the clay loam was ground into powder and then screened through a 2 mm sieve to remove large soil particles. Before sowing, all substrates were autoclaved (HVE-50 autoclave, Hirayama Manufacturing Corp., Japan) for 2 h. Except for the substrate, all other experimental operations and conditions were the same as those described for the general seed germination test.

To determine whether seeding depth affects seed germination, fine sand and clay loam substrate (consistent with the substrate used in the previous experiment) was added to a plastic box in a 7 cm-thick layer. Seeds were planted at depths of 0, 1.0, 3.0, and 5.0 cm. Thirty seeds were used in each of the four treatments, and each treatment was repeated three times. Germination was considered to have occurred with the germ broke through the top of the soil layer. All other experimental operations and conditions were the same as those described for the general seed germination test.

### Data analysis

IBM SPSS 19.0 was applied to analyze the variance and multiple comparisons of germination indexes of seeds under different treatments (Duncan’s new multiple range method). The significance levels of the above statistical analyses were set at *P* < 0.05.

## Results

### Morphological characteristics of seeds

The fruit of *H. hainanensis* is oval-shaped, 1.5 cm long, and formed of a capsule with a pedicel at the bottom. The pedicel is 8–10 cm long and grows on perennial branches, with 1–3 fruits on each peduncle (Fig. 1A). As the fruits mature, their color changes from green to yellow-green, they exhibit obvious ventral sutures, and they grow to a longitudinal diameter of 76.0 ± 6.3 mm and transverse diameter of 44.4 ± 2.7 mm. The fruits have a smooth and fleshy pericarp with a thickness of 4–5 mm (Fig. 1B and C). After the fruit is fully ripe, the pericarp splits longitudinally along the ventral suture into two petals, exposing the seed (Fig. 1D). The seed surface is coated with a yellow fleshy aril (Fig. 1D and E).

*H. hainanensis* seeds are ovoid or oval in shape, with a longitudinal diameter of 37.4 ± 4.0 mm, a transverse diameter of 22.0 ± 1.9 mm, a single seed weight of 6.8 ± 1.1 g, and a moisture content of 42.5% ± 3.4% (Table 1). The fresh seed coat is yellow-green, with irregular shallow furrows on the surface (Fig. 1F). The hilum is located at the larger end of the seed, covered with a light red long oval fleshy membrane, and the seed hole is located at one end of the long oval fleshy membrane, with light brown fleshy tissue around the hole (Fig. 1F). The seeds are rich in endosperm, with yellow-brown outer endosperm and white inner endosperm in a brown-white marbled pattern. The embryo is located on one side of the seed hole, irregularly shaped, and white; it is therefore difficult distinguish it from the endosperm (Fig. 1G and H). During germination, the radicle breaks through the seed coat from the seed hole and grows outward (Fig. 1I).

There was a significant difference in the fruit vertical diameter (FVD), fruit horizontal diameter (FHD), FVD/FHD ratio, seed vertical diameter (SVD), seed horizontal diameter (SHD), SVD/ SHD ratio, seed weight (SW), and moisture content (MC) among the three populations (*P* < 0.01). There was also a significant difference in fruit weight (FW) (*P* < 0.05) (Table 2). Among the three populations, the FVD, FVD/FHD, SVD, SW, and SMC were significantly greater in the XSBN population than in the other two populations. The FHD, FW, and SVD/SHD were not significantly different between the XSBN and NG populations but were significantly greater than those of the ND (Table 2).

**Table 2:** Morphological characteristics of the fruits and seeds of *H. hainanensis* in different populations

Population	ND	NG	XSBN	Mean	*P*
FVD (mm)	68.5 ± 0.7c	76.6 ± 0.9b	82.9 ± 1.4a	76.0	**
FHD (mm)	41.0 ± 1.2b	45.6 ± 0.8a	46.5 ± 0.9a	44.4	**
FVD/FHD	1.67 ± 0.03b	1.68 ± 0.03b	1.79 ± 0.02a	1.71	**
FW (g)	37.8 ± 1.2b	41.8 ± 3.8ab	44.0 ± 3.3a	41.2	*
SVD (mm)	35.5 ± 1.3b	34.2 ± 0.5b	42.6 ± 0.9a	37.4	**
SHD (mm)	23.2 ± 0.1a	19.6 ± 0.7b	23.4 ± 0.7a	22.0	**
SVD/SHD	1.54 ± 0.05b	1.76 ± 0.02a	1.83 ± 0.03a	1.71	**
SW (g)	6.4 ± 0.2b	5.8 ± 0.1b	8.3 ± 0.4a	6.8	**
SMC (%)	41.4 ± 1.2b	39.5 ± 1.0b	46.6 ± 1.7a	42.5	**

*Note* ND: Niandian Village, Daxin County, Chongzuo City, Guangxi Zhuang Autonomous Region; NG: Nonggang National Nature Reserve, Guangxi Zhuang Autonomous Region; XSBN: Xishuangbanna Botanical Garden in Yunnan Province; FVD: fruit vertical diameter; FHD: fruit horizontal diameter; FW: fruit weight; SVD: seed vertical diameter; SHD: seed horizontal diameter; SW: seed weight; SMC: seed moisture content. Data shown are the mean ± SE. Values followed by different letters in the same row indicate a significant difference in the same study object among the different treatments at *P* = 0.05. * indicates *P*<0.05 and ** indicates *P*<0.01

### Effects of seed biological characteristics on seed germination

#### Differences in seed germination among different populations

There were significant differences in GP, GE, and VI, but not in MGT, among the three populations of *H. hainanensis* Merr. (*P* < 0.05). The GP and VI of the seeds of the ND population were the highest, at 68.3% and 6.8, respectively. The GE values for the ND and ZWY populations was 38.3% and 36.7%, respectively, which was significantly higher than that of the NG population GE (11.7%) (Table 3).

**Fig. 2:**
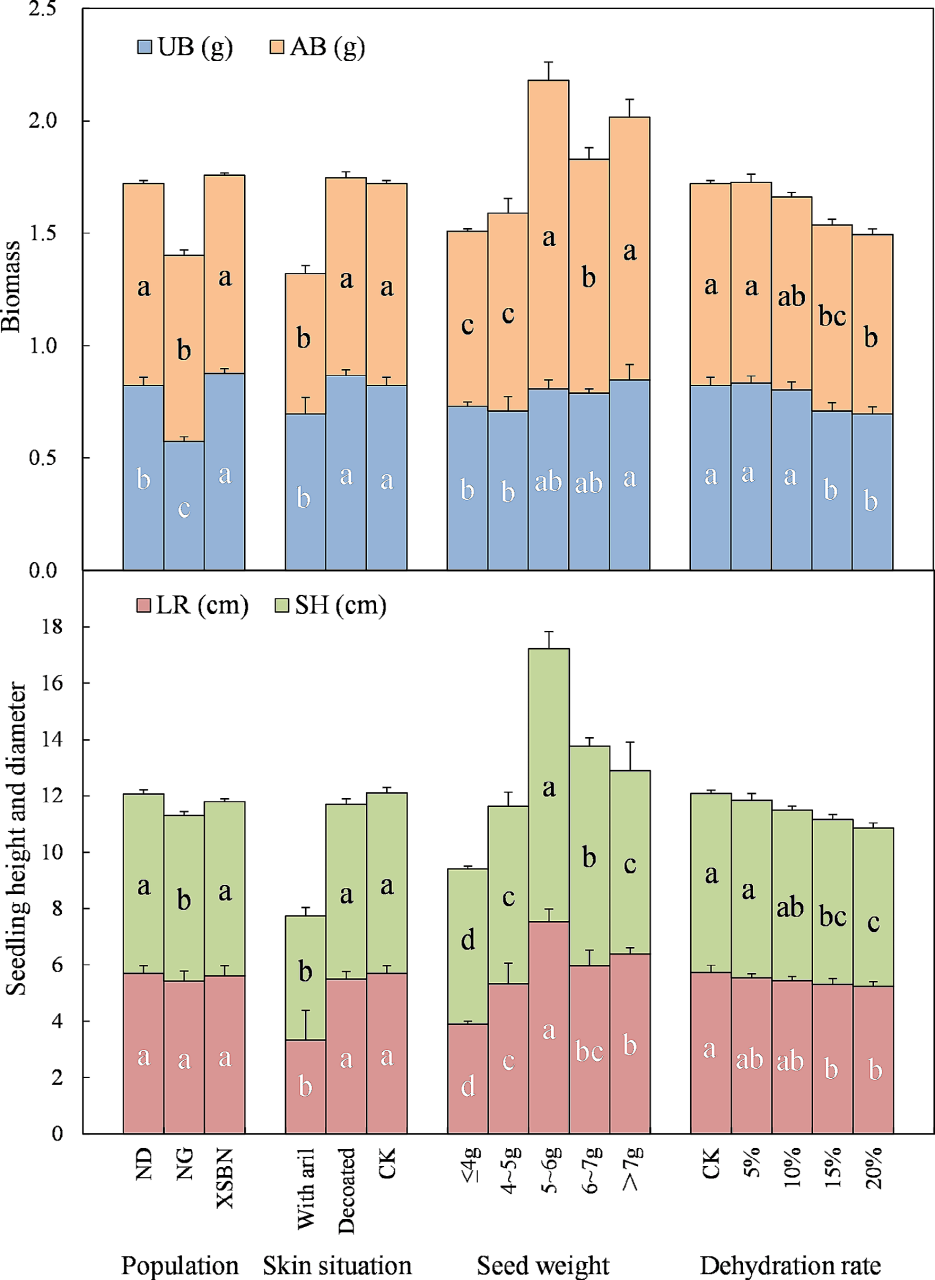
Effects of seed-factors on the growth and biomass of *H. hainanensis* seedlings. LR: length of root; SH: seedling height; UB: underground biomass; AB: aboveground biomass; ND: Niandian Village, Daxin County, Chongzuo City, Guangxi Zhuang Autonomous Region; NG: Nonggang National Nature Reserve, Guangxi Zhuang Autonomous Region; XSBN: Xishuangbanna Botanical Garden in Yunnan Province; WAS: seeds with aril and seed coat; DS: decorticated seeds; CK: aril removed and seed coat retained. Significant differences (*P* < 0.05, Duncan’s new complex difference method) are indicated by different letters. The error bars represented SD

There were significant differences in SH, AB, and UB, but not in LR, among the three populations of *H. hainanensis* Merr. (*P* < 0.05). The seeds UB of XSBN population was the largest, at 0.88 g. The AB and SH of the ND and XSBN populations were significantly higher than those of the NG population (Fig. 2).

#### Effects of aril and seed coat on seed germination

There were significant differences in GP, GE, MGT, and VI among the three treatments (*P* < 0.05). The GP and VI in CK treatment were the largest at 68.3% and 6.7%, respectively. Seeds from the CK and DS treatments showed significant higher GE than those that underwent the WAS treatment. DS treatment had a significant lower MGT than those from the WAS and CK treatments groups (Table 3).

There were significant differences in SH, LR, AB, and UB among the three treatments (*P* < 0.05). Seedlings from the CK and DS treatments had significant higher SH, LR, AB, and UB than those that underwent the WAS treatment (Fig. 2).

**Table 3:** Seed germination parameters of *H. hainanensis* under different seed-factor treatments

Study object	Treatment	GP	GE	MGT	VI
Population	ND	68.3 ± 2.9a	38.3 ± 2.9a	25.1 ± 0.3a	6.8 ± 0.3a
	XSBN	55.0 ± 5.0b	36.7 ± 2.9a	24.8 ± 0.3a	5.3 ± 0.4b
	NG	20.0 ± 5.0c	11.7 ± 2.9b	25.5 ± 0.7a	1.8 ± 0.4c
Pericarp	WAS	20.0 ± 5.0c	1.7 ± 2.9b	29.2 ± 1.0a	1.1 ± 0.2c
	DS	53.3 ± 5.8b	41.7 ± 2.9a	22.2 ± 0.3c	5.8 ± 0.6b
	CK	68.3 ± 2.9a	38.3 ± 2.9a	25.0 ± 0.3b	6.7 ± 0.2a
Seed weight	≤ 4 g	60.0 ± 5.0c	30.0 ± 0.0d	25.7 ± 0.9a	4.5 ± 0.2c
	4–5 g	70.0 ± 5.0b	48.3 ± 5.8b	23.4 ± 1.0b	7.1 ± 0.8b
	5–6 g	75.0 ± 5.0b	51.7 ± 2.9b	23.3 ± 0.6b	11.4 ± 0.1a
	6–7 g	86.7 ± 2.9a	68.3 ± 2.9a	22.0 ± 0.8b	11.2 ± 0.7a
	≥ 7 g	73.3 ± 2.9b	41.7 ± 2.9c	25.0 ± 0.3a	7.7 ± 0.7b
Dehydration rate	0%	68.3 ± 2.9a	38.3 ± 2.9a	25.0 ± 0.3b	6.8 ± 0.3a
	5%	70.0 ± 5.0a	38.3 ± 2.9a	25.1 ± 0.3b	6.8 ± 0.5a
	10%	38.3 ± 7.6b	21.7 ± 5.8b	25.7 ± 0.5ab	3.5 ± 0.6b
	15%	21.7 ± 2.9c	10.0 ± 0.0c	26.2 ± 0.9a	1.9 ± 0.2c
	20%	11.7 ± 2.9d	5.0 ± 0.0c	25.8 ± 0.3ab	1.0 ± 0.2d
	25%	0.0	–	–	–
	30%	0.0	–	–	–

*Note* GP: germination percentage; GE: germination energy; MGT: mean germination time; VI: vitality index; ND: Niandian Village, Daxin County, Chongzuo City, Guangxi Zhuang Autonomous Region; NG: Nonggang National Nature Reserve, Guangxi Zhuang Autonomous Region; XSBN: Xishuangbanna Botanical Garden in Yunnan Province; WAS: Seeds with aril and seed coat; DS: decorticated seeds; CK: seeds with aril removed and seed coat retained. Data shown are the mean ± SE. Different lower-case letters in the same column indicate significant differences at *P* = 0.05, whereas the same lower-case letters indicate non-significant differences. – indicates no data

#### Effects of seed weight on seed germination

**Fig. 3:**
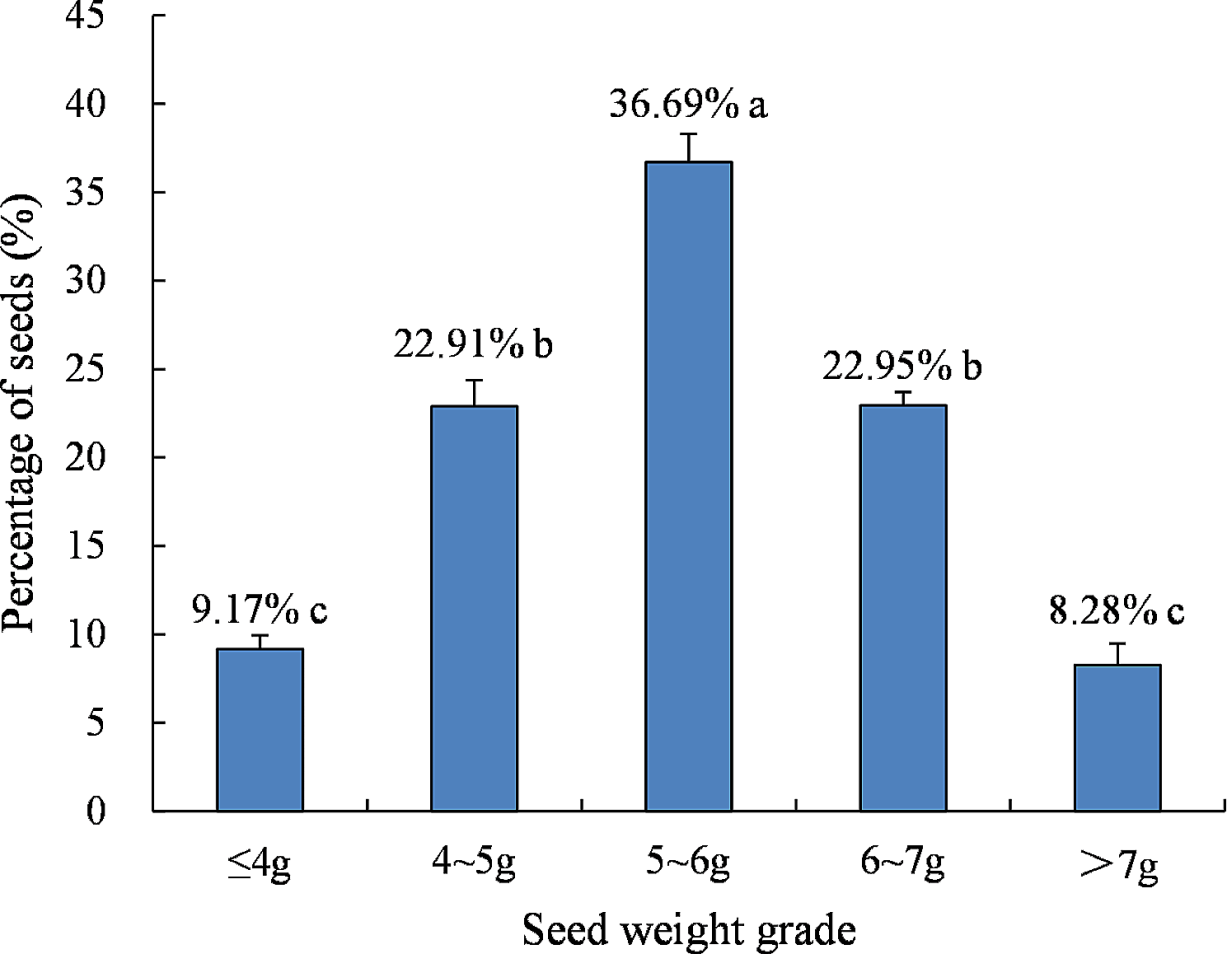
Percentage of seeds in the five seed weight grades in *H. hainanensis*. Significant differences (*P* < 0.05, Duncan’s new complex difference method) are indicated by different letters

There was a significant difference in the distribution of seeds among the five seed weight grades (*P* < 0.01, Fig. 3). The largest proportion of the seeds were in the 5–6 g category (36.69%), significantly higher than the proportions of seeds distributed in the other four weight grades. Seeds ≤ 4 g and > 7 g accounted for the smallest proportions, at 9.17% and 8.28%, respectively.

There were significant differences in GP, GE, MGT, and VI among the five seed weight grades (*P* < 0.05). Of these, the GP and GE of 6–7 g seeds were the largest, at 87.9% and 68.3%, respectively, and the VI of 5–6 g and 6–7 g seeds were 11.4, and 11.2 respectively, which were significantly higher than those of the other three weight grades. The MGTs of seeds in the 4 ~ 5 g, 5 ~ 6 g and 6 ~ 7 g were 23.4 d, 23.3 d and 22.0 d, respectively, which were significantly shorter than the MGTs of the seeds in the ≤ 4 g and > 7 g weight grades (Table 3).

**Fig. 4:**
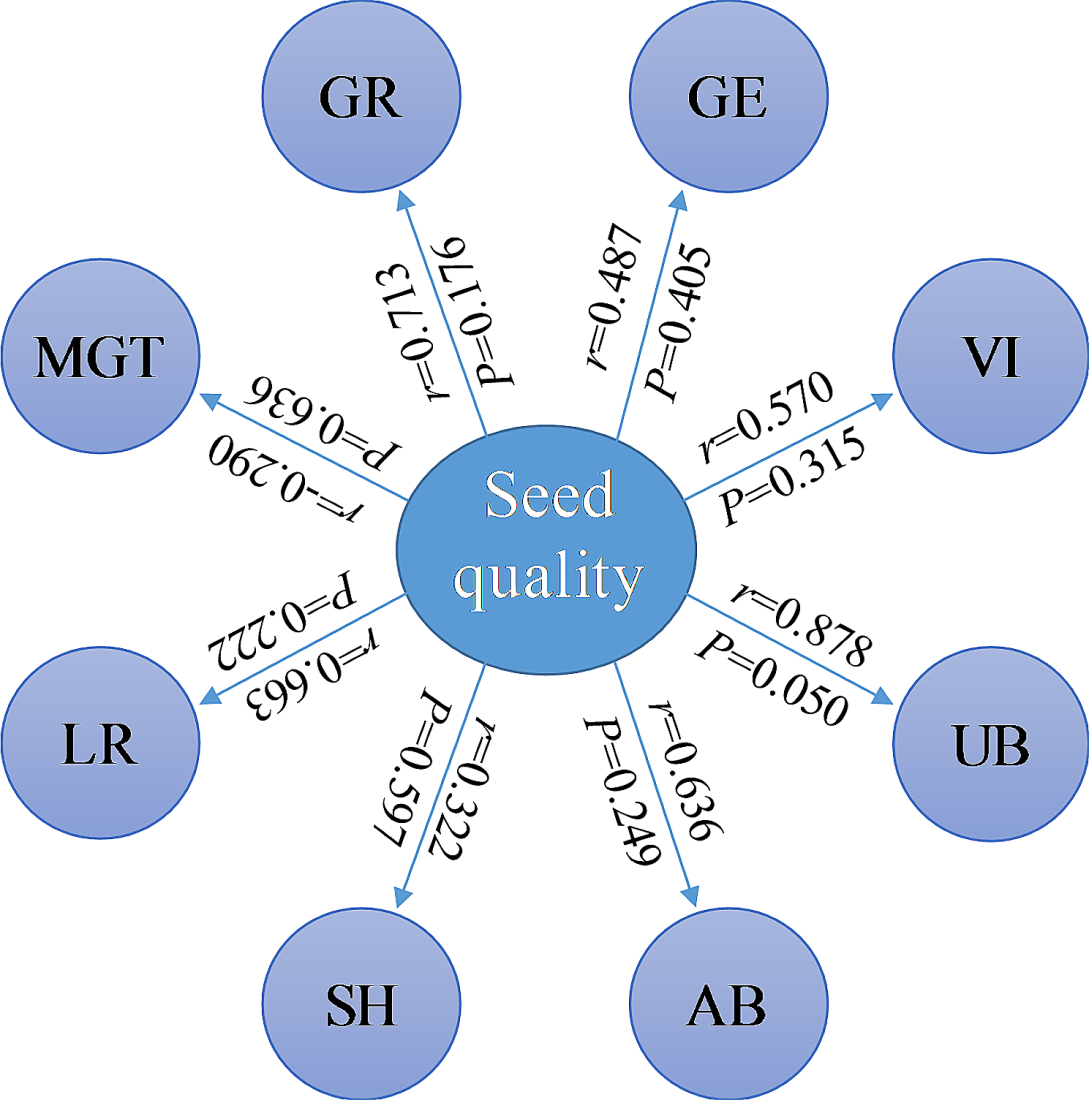
Correlation between seed weight and seed germination and seedling growth indexes of *H. hainanensis*. GP: germination percentage; GE: germination energy; MGT: mean germination time; VI: vitality index; LR: Length of root; SH: seedling height; UB: underground biomass; AB: aboveground biomass; *r*: correlation coefficient

There were significant differences in SH, LR, AB, and UB among the five seed weight grades (*P* < 0.05). The SH and LR of seedlings germinated from 5 to 6 g seeds were the largest among the five seed weight grades, while the AB of seedlings germinated from 5 to 6 g seeds and seeds > 7 g was significantly higher than that of seedlings germinated from seeds ≤ 4 g, 4–5 g, and 6–7 g seeds, and the UB of seedlings germinated from 5 to 6 g, 6–7 g, and > 7 g seeds was significantly greater than that of seedlings germinated from ≤ 4 g and 4–5 g seeds (Fig. 2),

On the whole, among the five seed weight grades, 6–7 g seeds germinated better and seedlings germinated from 5 to 6 g seeds grew better. Correlation analysis showed the seed weight was not significantly correlated with seed germination and seedling growth indicators (Fig. 4).

#### Effects of seed dehydration rate on seed germination

**Fig. 5:**
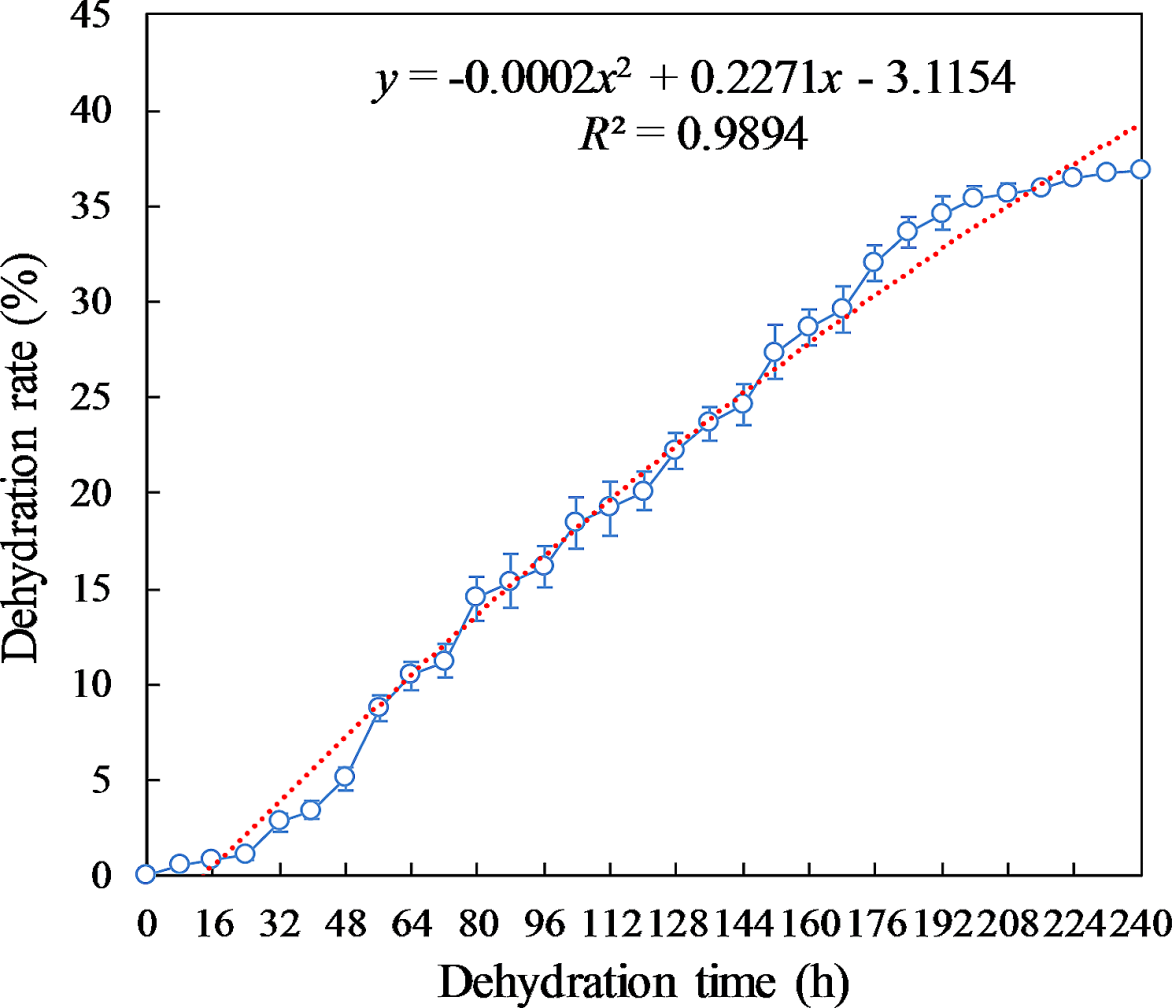
Dehydration rate of *H. hainanensis* seeds over time. *R*^2^: fitting coefficient. The error bars represented SD

The dehydration rate of *H. hainanensis* seeds gradually increased with the duration of dehydration and was distributed in a binomial model. The dehydration rate of the seeds was slow from 0 to 24 h, fast from 240 to 192 h, and stable after 192 h. The dehydration rate reached 36.87% at 240 h (Fig. 5).

Among the five treatments (CK, 5%, 10%, 15%, and 20%), there were significant differences in GP, GE, and VI (*P* < 0.05), but there was no significant difference in MGT (*P* > 0.05). There was no significant difference in the seed germination index between seeds with a 5% dehydration rate and those under the CK treatment. With the continuous increase in dehydration rate, the GP, GE, and VI of the seeds decreased continuously. At a dehydration rate of 20%, the GP, GE, and VI of the seeds were 11.7%, 5.0%, and 1.0, respectively, which were significantly lower than those of seeds from the CK treatment (Table 3).

Among the five treatments, there were significant differences in SH, AB, and UB, but there was no significant difference in LR. There was no significant difference in the seedling growth index between seeds with a 5% dehydration rate and those under the CK treatment. With the continuous increase in dehydration rate, the SH, AB, and UB of the seedlings decreased continuously. At a dehydration rate of 20%, the SH, AB, and UB of the seedlings were 5.6 cm, 0.80 g, and 0.70 g, respectively, which were significantly lower than those of seedlings from the CK treatment (Fig. 2).

### Effects of environmental factors on seed germination

#### Effects of temperature and light on seed germination

The seeds had greater GPs at 30 and 30/25°C, 63.3% and 68.3%, respectively, and the GE of seeds was the highest at 35 and 35/25°C, both 43.3%. There was no significant difference in VI among seeds at 30, 35, 30/25, and 35/25°C, but was significantly higher than that at 25 and 40 °C (Table 4). These results indicated that the most suitable germination temperature range for *H. hainanensis* seeds was 30–35 °C.

**Fig. 6:**
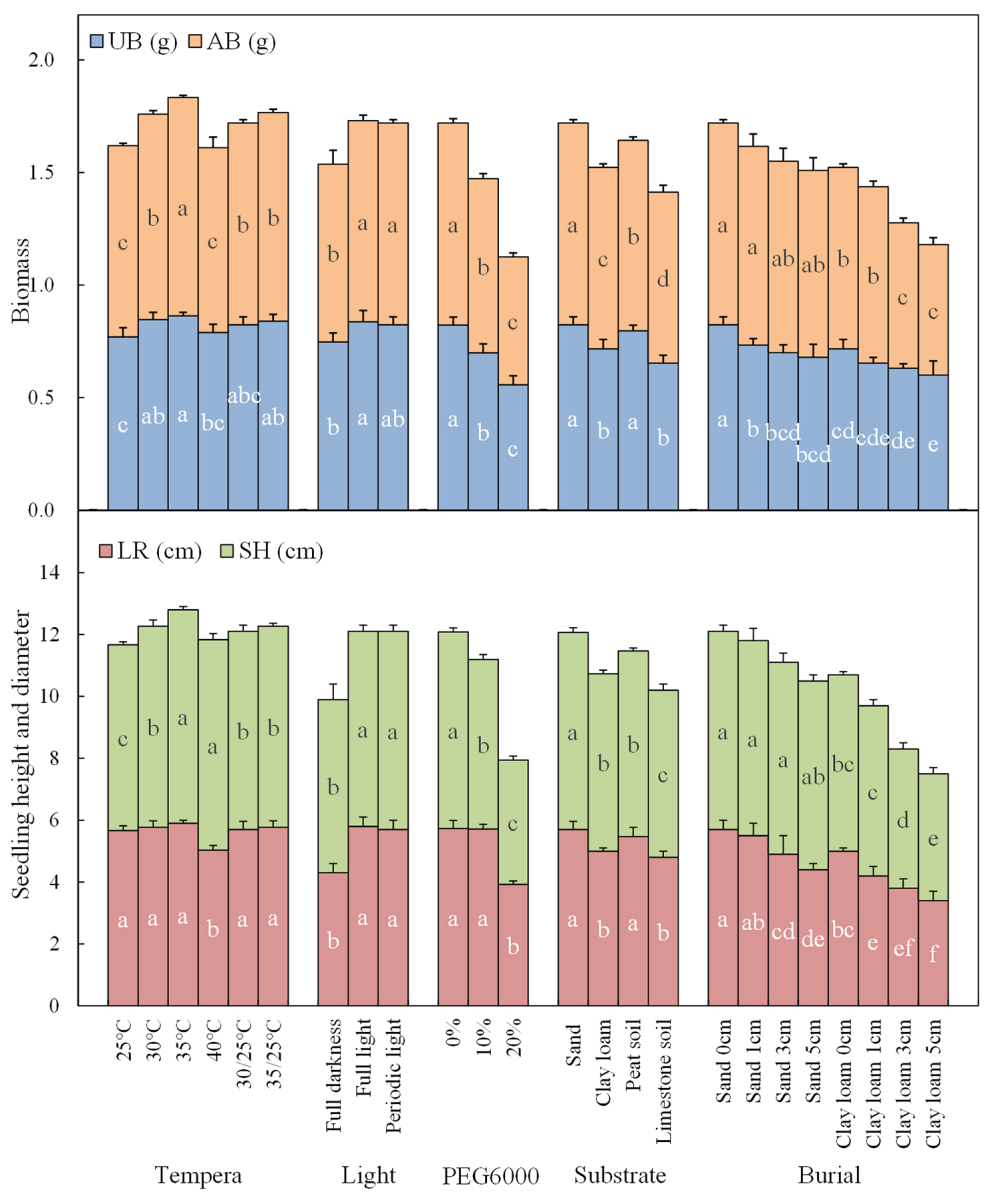
The growth and biomass of *H. hainanensis* seedlings under different environmental conditions. LR: length of root; SH: seedling height; UB: underground biomass; AB: aboveground biomass. Significant differences (*P* < 0.05, Duncan’s new complex difference method) are indicated by different letters. The error bars represented SD

The SH was largest at 35 and 40 °C, at 6.9 and 6.8 cm, respectively, which was significantly greater than that at the other temperatures. The LR did not vary significantly at 25, 30, 35, 30/25, and 35/25°C, at which temperatures it was greater than that at 40 °C. The AB and UB were greater at 35 °C, 0.97 and 0.86 g, respectively (Fig. 6). These findings indicated that 35 °C was the most suitable temperature for the growth of *H. hainanensis* Merr. seedlings.

There was no significant difference in GP among seeds under the three light treatments (*P* > 0.05) (Table 4). There was no significant difference in SH, LR, AB, and UB between seeds under the full light and periodic light treatments, all of which were significantly higher than those for seeds under the full darkness treatment (Fig. 6).

**Table 4:** Seed germination parameters of *H. hainanensis* under different environmental conditions

Study object	Treatment	GP	GE	MGT	VI
Temperature (℃)	20	0.0	-	-	-
	25	48.3 ± 2.9c	13.3 ± 2.9c	28.7 ± 0.4a	4.0 ± 0.3c
	30	63.3 ± 2.9ab	36.7 ± 2.9b	25.5 ± 0.8b	6.3 ± 0.4a
	35	58.3 ± 5.8b	43.3 ± 2.9a	22.2 ± 1.7c	6.9 ± 0.2a
	40	41.7 ± 2.9c	36.7 ± 2.9b	20.2 ± 0.3d	5.0 ± 0.5b
	30/25	68.3 ± 2.9a	38.3 ± 2.9ab	25.0 ± 0.3b	6.7 ± 0.2a
	35/25	60.0 ± 5.0b	43.3 ± 2.9a	22.0 ± 0.2c	6.9 ± 0.8a
Light	Full darkness	66.7 ± 2.9a	–	–	–
	Full light	65.0 ± 5.0a	–	–	–
	Periodic light	68.3 ± 2.9a	–	–	–
PEG6000	0%	66.7 ± 2.9a	36.7 ± 2.9a	25.0 ± 0.2b	6.6 ± 0.4a
	10%	63.3 ± 2.9a	35.0 ± 0.0a	25.7 ± 0.2b	5.7 ± 0.2b
	20%	35.0 ± 5.0b	15.0 ± 5.0b	26.9 ± 0.9a	2.1 ± 0.4c
	30%	0.0	-	-	-
Substrate	Sand	68.3 ± 2.9a	38.3 ± 2.9a	25.0 ± 0.3b	6.7 ± 0.2a
	Clay loam	40.0 ± 5.0c	20.0 ± 0.0c	27.1 ± 1.0a	3.3 ± 0.3c
	Peat soil	66.7 ± 2.9a	38.3 ± 2.9a	25.3 ± 0.1b	6.2 ± 0.2a
	Limestone soil	56.7 ± 2.9b	33.3 ± 2.9b	25.1 ± 0.2b	4.7 ± 0.5b
Burial depth	Sand 0 cm	68.3 ± 2.9a	38.3 ± 2.9a	25.0 ± 0.3e	6.7 ± 0.2a
	Sand 1 cm	43.3 ± 2.9b	8.3 ± 7.6c	30.1 ± 1.0c	3.5 ± 0.2b
	Sand 3 cm	25.0 ± 5.0d	0.0 ± 0.0d	31.1 ± 0.6bc	2.0 ± 0.6c
	Sand 5 cm	18.3 ± 2.9e	0.0 ± 0.0d	32.6 ± 1.3ab	1.2 ± 0.2d
	Clay loam 0 cm	40.0 ± 5.0b	20.0 ± 0.0b	27.1 ± 1.0d	3.3 ± 0.3b
	Clay loam 1 cm	31.7 ± 2.9c	0.0 ± 0.0d	32.2 ± 0.5ab	2.0 ± 0.2c
	Clay loam 3 cm	23.3 ± 2.9de	0.0 ± 0.0d	32.4 ± 1.2ab	1.2 ± 0.1d
	Clay loam 5 cm	11.7 ± 2.9f	0.0 ± 0.0d	33.2 ± 1.1a	0.5 ± 0.2e

*Note* GP: germination percentage; GE: germination energy; MGT: mean germination time; VI: vitality index. Data shown are the mean ± SE. Different lower-case letters in the same column indicate significant differences at *P* = 0.05, whereas the same lower case letters indicate non-significant differences. – indicates no data

#### Effects of drought on seed germination

There were significant differences in GP, GE, VI, and MGT among the different drought treatments (*P* < 0.05). The GP, GE, and MGT were not significantly different between seeds treated with PEG6000-10% and those treated with PEG6000-0%. The GP and GE of seeds treated with PEG6000-10% or PEG6000-0% were significantly higher than of seeds treated with PEG6000-20%, while MGT was significantly shorter than under the PEG6000-20% treatment. The VI of seeds treated with PEG6000-0% was significantly higher than of those treated with PEG6000-10% or PEG6000-20% (Table 4).

There were significant differences in SH, LR, UB, and AB among the different drought treatments (*P* < 0.05). The LR was not significantly different between seedlings grown from seeds treated with PEG6000-10% or PEG6000-0% but was greater than the LR of seedlings grown from PEG6000-20%-treated seeds. The SH, AB, and UB from seeds treated with PEG6000-0% were significantly higher than from those treated with PEG6000-10% or PEG6000-20% (Fig. 6).

#### Effects of substrate on seed germination

There were significant differences in the seed germination parameters among the four different substrates (*P* < 0.05). The GP, GE, and VI of seeds sown in fine sand and peat soil substrates were higher than those for seeds sown in clay loam and limestone soil. The MGT of seeds sown in the clay loam substrate was the longest (27.1 days) and significantly higher than that for seeds sown in the other three substrates (Table 4).

There were significant differences in the seedling growth indexes among the four different substrates (*P* < 0.05). The LR and UB of seedlings in fine sand and peat soil substrates were higher than those for seedlings in clay loam l and limestone soil. The SH and AB of seedlings grown in the fine sand substrate were the highest and significantly greater than those for seedlings in the other three substrates (Fig. 6).

#### Effects of burial depth on seed germination

The GP, GE, and VI of seeds in the two substrates decreased with the increase in burial depth, while the MGT increased with the increase in burial depth. At the same burial depth, the GP, GE, and VI of seeds in the fine sand matrix were significantly higher than those of seeds in the clay loam substrate, while the MGT in fine sand was lower than that of seeds in the clay loam substrate (Table 4).

The SH, LR, AB, and UB of seedlings in the two substrates decreased with the increase in burial depth. At the same burial depth, the SH, LR, AB, and UB of seedlings in the fine sand matrix were significantly higher than those of seedlings grown in the clay loam substrate (Fig. 6).

## Discussion

### Fruit characteristics and ecological adaptability of *H. Hainanensis*

Seed morphology not only determines the dispersal ability of a species, but also affects seed germination and seedling colonization, which in turn affects the distribution pattern of the resulting populations [21, 22]. Adult *H. hainanensis* trees are about 15–30 m tall, with large fruits that fall easily from the mother plant after ripening. The smooth, hard, and thick pericarp (4–5 mm) can not only reduce the impact with the ground to protect the internal seeds from damage, but also prevent the seeds from being eaten by insects, ants, and birds. The seeds are exposed after the fruits crack open, and the yellow fleshy aril outside the seeds can attract animals to feed on them, which can increase their dispersal distance. However, the pericarp and fleshy aril rot easily under conditions of high temperature and high humidity, and this is toxic to the seeds. Moreover, fruits and seeds rely on gravity for dispersal and are distributed in aggregation [15], which can intensify the spread of toxicity and thus is not conducive to seed germination. Therefore, the pericarp and aril of *H. hainanensis* provide seed protection and assist in seed dispersal, but can also pose risks to seeds.

There are two strategies for input to seed resources: One is to produce a small number of large seeds to gain a competitive advantage; and the other is to produce many small seeds to occupy more ecological niches [23]. *H. hainanensis* seeds (with a longitudinal diameter of 37.4 mm, a transverse diameter of 22.0 mm, and a single seed weight of 6.8 g) are larger than *Horsfieldia pandurifolia* seeds (with a longitudinal diameter of 33.2 mm, a transverse diameter of 19.2 mm, and a single seed weight of 5.91 g) and smaller than *Horsfieldia tetratepala* seeds (with a longitudinal diameter of 40.6 mm, transverse diameter of 23.9 mm, and single seed weight of 10.57 g) [24]. In fact *H. hainanensis* seeds have a higher than average seed weight of for arboreal species, with the average being 0.328 g [25]. Therefore, *H. hainanensis* prefers the reproductive strategy of producing a small number of large seeds to support rapid early seedling growth through a well-developed endosperm structure to gain a competitive advantage.

The eco-geographical factors of seed origin, such as latitude, longitude, altitude, moisture, nutrition, and surrounding vegetation species and their degree of cover and level of interference, lead to variation and differentiation in plant genetic material during the process of environmental adaptation, affecting the variation of offspring through maternal effects. This variation is often exhibited in the early stage of the offspring life cycle (seed and seedling stage) [26]. In this study, the morphological size and weight of fruits and seeds of the XSBN population were larger than those of the other two populations, which may be due to the large variation of fruits and seeds, owing to the long distance between the XSBN population and ND and NG populations and the large difference in environmental conditions. However, by comparing the seed germination index and seedling growth index of the three populations, it was found that there were great differences in seed GP and seedling growth between the ND and NG populations, which grew close together under similar environmental conditions. It may be that seed germination and seedling growth in the same environment are mainly affected by self-factors such as seed development, health, and genetic information [27].

### Factors affecting seed germination and seedling growth in *H. Hainanensis*

The effects of the aril and seed coat on seed germination are mainly associated with mechanical obstruction and chemical inhibition. Mechanical obstruction mainly hinders seed water absorption and gas exchange, which inhibits seed respiration, weakens internal metabolic reactions, and affects seed germination. Components such as oil, abscisic acid, organic acid, alkaloids and phenols in the aril and seed coat affect seed germination by hindering seed water absorption, inhibiting respiration, inhibiting enzyme activity, changing the osmotic pressure, and hindering embryo growth [28, 29]. The GP and VI of *H. hainanensis* seeds from the DS treatment were significantly lower than those of CK seeds. There was no significant difference in seedling growth and biomass between the DS and CK treated seeds, indicating that the seed coat had no inhibitory effect on seed germination and early seedling growth. On the contrary, retaining the seed coat was conducive to seed germination, as the seed coat can protect seeds from environmental factors [30]. The GP, VI, seedling growth, and biomass of seeds following the WAS treatment were significantly lower than those of CK seeds, indicating that the aril had a significant inhibitory effect on seed germination and early seedling growth. However, as the aril does not completely encapsulate the seed in this species (Fig. 1E), it does not hinder seed water uptake and gas exchange. However, the aril in the seed of *H. hainanensis* is fleshy and easily infected by mildew [15]. Therefore, we speculate that the inhibition of seed germination and seedling growth by the aril may be caused by internal inhibitors or mildew toxicity, or both. The specific reasons need to be further studied [31].

It has been noted that seed size is positively correlated with germination rate, GP, and seedling growth [32, 33]. However, it is also reported that seed size is not significantly correlated with germination [34, 35]. The seed size was not significantly correlated with seed germination parameters and seedling growth indexes of *H. hainanensis* in this study, consistent with results of seed studies on *Garcinia paucinervis* [20], *Cirsium pitcheri* [36], and *Leymus racemosus* [35]. *H. hainanensis* mainly produce medium-sized seeds (5–6 g). The GP of medium-sized seeds was 75.0%, second only to 6–7 g seeds, and the seedlings grew well. Therefore, the production of larger seeds may be an adaptive mechanism in *H. hainanensis* even though they are difficult to spread and more prone to predation. Although small seeds are easier to spread and can escape predation [37], the GP for small seeds in *H. hainanensis* was relatively low. Therefore, *H. hainanensis* chooses a breeding strategy that produces more medium-sized seeds, which not only increases the seed spreading distance, but also ensures a higher GP and seedling quality to promote population reproduction, similar to the population reproduction strategies of *Dalbergia sissoo* and *Acacia catechu* [38, 39].

Recalcitrant seeds are generally those that have a high water content at dispersal, maintain metabolic activity, and are sensitive to dehydration and low temperatures [40]. In this study, the initial MC of the *H. hainanensis* seeds was high, at 41.4%, with a GP of 68.3%. Under natural conditions, the GP gradually decreased as the seed dehydration rate gradually increased. At the seed dehydration rate of 5%, the seed GP decreased insignificantly. However, after 64 h, the seed dehydration rate exceeded 10% and the seed GP decreased sharply, indicating that *H. hainanensis* seeds can only tolerate mild dehydration. After 144 h of dehydration, the dehydration rate of seeds exceeded 25%, the seeds were completely inactivated, and the GP was 0, indicating that the seeds have a short life span and are sensitive to dehydration. Furthermore, according to the experiments on the effects of temperature on seed germination, the seeds did not germinate at 20 °C (Table 4), suggesting that *H. hainanensis* seeds are also sensitive to low temperatures. Therefore, *H. hainanensis* seed behavior is similar to that of other rainforest species such as such as *Hopea mollissima* [41], *Butia capitate* [42], and *Garcinia mangostana* [43]. The *H. hainanensis* seeds were recalcitrant and belong to the highly recalcitrant type proposed by Farrant [40].

Temperature is crucial to seed germination, and the appropriate temperature can enhance enzyme activity, promote material and energy conversion, and facilitate seed germination [44]. The most suitable temperature range for the germination of *H. hainanensis* seeds is 30–35 ℃, and the most suitable temperature for seedling growth is 35 ℃, indicating that *H. hainanensis* seeds prefer high temperatures from germination to the growth and development stage, which is consistent with the humid tropical rainforest habitat in which they are planted. Notably, variable temperature conditions can significantly enhance seed germination in some plants, as the interconversion of high and low temperatures can promote the physiological activities of various enzyme systems within the seed, resulting in seed coat shrinkage and facilitating the exchange of gases between the seed and the outside world [45, 46]. There was no significant difference in the seed germination parameters and seedling growth indexes of *H. hainanensis* seeds at 30 and 30/25°C and no significant difference in seed germination parameters, LR, and UB of seeds at 35 and 35/ 25 °C. The SH and AB of seeds at 35 °C were, however, significantly higher than those of seeds at 35/25°C. Therefore, variable temperature conditions do not promote seed germination in this species, and a constant temperature is more suitable for the growth of seedlings than variable temperature, which was consistent with the response of seed germination to temperature of *Orobanche* [47] and *Trichocereus terscheckii* [48]. This may be because the internal biochemical reactions in seeds during germination is not strictly dependent on temperature, but high temperature is needed to promote metabolic activity during seedling growth [49]. The most suitable germination temperature for seeds is closely related to climate change in their habitat. A constant temperature of 30 ℃ or a variable temperature of 30 ℃/25 ℃ was most suitable for the germination of *H. hainanensis* seeds, which is an adaptation to their habitat. *H. hainanensis* seeds mature in mid-May, during the rainy season in tropical rainforest. During this period, the day and night temperatures are between 25 ℃ and 30 ℃, which, along with sufficient water, promote the physiological activities of various enzyme systems within the seeds, preparing them for germination. After 20–25 days of cellular activity, the seeds begin to germinate, when the external temperature rises to around 35 ℃, which is suitable for the growth of seedlings. This germination mechanism ensures that most seeds germinate at the appropriate time, increasing the chances of seedling survival.

Based on the effect of light on seed germination, seeds are classified as light-demanding, light-neutral, or light-abstaining [50]. In this study, there was no significant difference in GP among seeds under three light treatments (full darkness, periodic light, and full light), indicating that light has no significant effect on seed germination in *H. hainanensis.* The seeds of this species are light neutral, which may be the reason *H. hainanensis* is distributed in the tropical rain forest of low-altitude limestone mountains, where the light environment under the forest canopy is complex and changeable. Furthermore, its fruits are oval, and most of them fall in places with weak light, such as stone crevices and depressions near the mother plant, or where light is blocked by leaf litter. In order to adapt to this light environment, *H. hainanensis* seeds have adopted a light-insensitive germination strategy. The SH, LR, AB, and UB of seedlings grown from seeds under full light and periodic light treatments were significantly higher than those of seeds under the full darkness treatment, indicating that light promoted the early growth of seedlings, probably due to the seedlings’ ability to carry out photosynthesis and accumulate more organic matter under light conditions [51].

Water is the main environmental factor affecting seed germination and seedling growth [52]. According to this study, there was no significant difference in GP, GE, and MGT between those under mild drought stress and those under the CK treatment. However, the VI, SH, AB, and UB were significantly lower than those of seeds and seedlings under the CK treatment, indicating that seed germination can tolerate mild drought. Seedling growth was more sensitive to drought and could be significantly inhibited by even mild drought. Under moderate drought stress, *H. hainanensis* exhibited significantly decreased seed GP, GE, VI, and seedling growth index values, and significantly increased MGT. Under severe stress, the seeds did not even germinate, indicating that drought stress can significantly inhibit seed germination and seedling growth. This may occur because water deficiency inhibits internal enzyme activities and material conversion in seeds, which in turn affect physiological metabolic activities and reduce seed vitality [53, 54].

Among the four growth substrates, the GP, GE, and VI of seeds sown in clay soil and soil substrates from rocky mountain areas were significantly lower than those of seeds sown in fine sand and peat soil substrates, suggesting that *H. hainanensis* seeds germinate well in fine sand and peat soil, whereas and clay soil and soil from rocky mountain areas were not conducive to germination. This may be due to the poor aeration and water permeability of clay loam and rocky mountain areas soil, where seeds are prone to produce harmful intermediates and excessive respiratory heat under anoxic conditions, resulting in reduced seed viability, while fine sand and peat soils are loose and highly permeable, providing an adequate oxygen supply, which reduces the occurrence of mildew and is favorable to seed germination [20, 55]. The LR and UB of seedlings were larger in those grown in fine sand substrate, and the SH and AB of seedlings in the fine sand substrate were the largest, indicating that fine sand is more suitable for growing *H. hainanensis* seedlings. Fine sand has better permeability than clay soil. In this study, with the increase in seed burial depth, the seed germination time, GP, VI, seedling growth, and biomass index decreased gradually, while the MGT increased gradually, and the seeds in fine sand substrate germinated better than in clay soil at the same depth. These findings indicated that seed germination and seedling growth are sensitive to substrate aeration and water permeability. The deeper the seed burial, the worse the aeration, and the lower the oxygen concentration, the more inhibited the seed germination and seedling growth [20].

### The causes of *H. Hainanensis* endangerment based on seed germination and potential protection strategies

The presence of any factors that are not conducive to seed germination at the seed germination stage can have a direct impact on the life history strategy of plant reproduction, the stability of the population, and the generation and supplementation of new individuals, resulting in the endangerment or restricted distribution of the species [20, 56]. The fruits of *H. hainanensis* are larger than most and spread by means of gravity. After fruit falls, the aggregated seeds are distributed in an area 2 ~ 3 m from the mother plant, a phenomenon that increases the competition between seeds. If there is a lack of animal handling and rain wash, mildew grows on arils under high temperature and high humidity conditions causing toxicity to seeds. Seed aggregate distribution aggravates the spread of toxins and impacts seed germination. Even if seeds germinate successfully to form seedlings, due to the limited light conditions near the mother plant, the seedlings do not get enough light, so it is difficult to gain a dominant position in competition with other plants. In addition, the natural populations of *H*. *hainanensis* are mostly distributed in rocky mountain areas where there is a high degree of rock exposure. If seeds fall on rocks with poor water retention, they dehydrate, which influences their lifespan. Substrates with good water retention and permeability are therefore favorable for seed germination and seedling growth in *H. hainanensis*. Nevertheless, the soil found in its distribution area is limestone soil. Although limestone soil offers strong water retention, its ventilation and water permeability are poor. Water conditions fluctuate widely in rocky maintain areas, leading quickly to soil drought, which inhibits seed germination and seedling growth. Thus, due to the seed transmission method used by *H. hainanensis*, the aril leaves seeds susceptible to dehydration and drought and vulnerable to environment factors like light, soil, and water, which may be an important reason for the low seed germination rate seen in this species and the scarcity of seedlings in its populations.

Based on these findings, we suggest that fallen fruits of *H. hainanensis* are collected artificially to reduce the fallen-fruit density, and that fruits that fall on rocks are collected and the seeds (i) sown in an environment with good water retention and breathability outside the mother plant canopy, or (ii) returned to an indoor environment for artificial cultivation of seedlings. At the appropriate time, the seedlings could be reintroduced to the environment to meet the needs of population renewal. We can artificially strengthen soil porosity and light transmittance in a *H. hainanensis* forests to improve seedling growth and create conditions that enable the natural renewal of individuals in the population. Although we have studied the biological characteristics of *H. hainanensis* seeds and the effects of these features and environmental factors on seed germination and provided a theoretical basis for the mechanisms underlying this species’ endangerment, along with suggestions for the protection of germplasm resources and artificial breeding, there are still many questions remaining. For example, the mechanism by which the aril inhibits seed germination, the features that underpin the sensitivity of seeds to dehydration, and the effects of animals, plants, and litter in the source habitat require further study. Therefore, the population renewal mechanism of *H. hainanensis* should be further investigated.

## Conclusions

*H. hainanensis* fruits are capsules where in the fleshy aril and pericarp wrap around the seeds, providing protection and assisting seed dispersal, but also posing risks to the seeds. The seeds of *H. hainanensis* have a higher average seed weight than many arboreal species, with a longitudinal diameter of 37.4 mm, transverse diameter of 22.0 mm, and single grain weight of 6.8 g. *H. hainanensis* populations tend to produce a small number of large seeds to gain a competitive advantage. There were significant differences in seed morphology and germination characteristics among different populations, and the seed quality of populations in Niandian village, Daxin County, Chongzuo City was better. The seed aril significantly inhibits seed germination, whereas the seed coat does not inhibit seed germination. The germination percentage of seeds with a single seed weight of 6 ~ 7 g was higher, and the seedlings originating from 5 ~ 6 g seeds grew better. *H. hainanensis* seeds are sensitive to dehydration; after 144 h of dehydration, the dehydration rate of the seeds exceeded 25%, and they were completely inactivated. In addition, *H. hainanensis* seeds are not tolerant to drought and low temperature, with drought significantly inhibiting seed germination and seedling growth. Seeds did not germinate at 20 ℃. Therefore, *H. hainanensis* seed is a typical recalcitrant seed, which is suitable for germination on the surface of a moist substrate, such as fine sand or peat soil, with good water retention and air permeability at 30–35 °C. The biological characteristics of seed and environmental factors limit the germination of *H. hainanensis* seeds, which is an important reason for their difficulty in regeneration. The results of this study are not only helpful to further analyses of the reasons for *H. hainanensis* endangerment, but also provide a scientific basis for the artificial cultivation of this species.

## Data Availability

The data that support the findings of this study are available from the corresponding author upon reasonable request.

## Abbreviations

ND: Niandian village, Daxin County, Guanxi
NG: Nonggang Nature Reserve in Guangxi
XSBN: Xishuangbannan Botanical Garden in Yunnan
FVD: Fruit vertical diameter
FHD: Fruit horizontal diameter
FW: Fruit weight
SVD: Seed vertical diameter
SHD: Seed horizontal diameter
SW: Seed weight
SMC: Seed moisture content
WAS: Seeds with aril and seed coat
DS: Decorticated seeds
CK: Aril removed and seed coat retained
GP: Germination percentage
GE: Germination energy
MGT: Mean germination time
VI: Vitality index
LR: Length of root
SH: Seedling height
UB: Underground biomass
AB: Aboveground biomass
